# Abrogation of type-I interferon signalling alters the microglial response to Aβ_1–42_

**DOI:** 10.1038/s41598-020-59917-0

**Published:** 2020-02-21

**Authors:** Zachery Moore, Frank Mobilio, Frederick R. Walker, Juliet M. Taylor, Peter J. Crack

**Affiliations:** 1 Department of Pharmacology and Therapeutics, The University of Melbourne, Melbourne, Australia; 2 School of Biomedical Sciences and Pharmacy, The University of Newcastle, Callaghan, Australia

**Keywords:** Cellular neuroscience, Microglia

## Abstract

Neuroinflammation and accompanying microglial dysfunction are now appreciated to be involved in Alzheimer’s disease (AD) pathogenesis. Critical to the process of neuroinflammation are the type-I interferon (IFN) family of cytokines. Efforts to phenotypically characterize microglia within AD identify distinct populations associated with type-I IFN signalling, yet how this affects underlying microglial function is yet to be fully elucidated. Here we demonstrate that Aβ_1–42_ exposure increases bioactive levels of type-I IFN produced by primary microglia alongside increased expression of type-I IFN related genes. Primary microglia isolated from brains of APP_swe_PS1_ΔE9_ mice with ablated type-I IFN signalling show an increased phagocytic ability to uptake FITC-Aβ_1–42_. Correlative assessment of plaque sizes in aged APP_swe_PS1_ΔE9_ mice with abrogated type-I IFN signalling show unchanged deposition levels. Microglia from these mice did however show alterations in morphology. This data further highlights the role of type-I IFN signalling within microglia and identifies a role in phagocytosis. As such, targeting both microglial and global type-I IFN signalling presents as a novel therapeutic strategy for AD management.

## Introduction

Alzheimer’s disease (AD) is now recognised as the most common form of dementia and is now estimated to be the 5^th^ largest cause of death globally^1^. AD has been classically characterised by its two hallmark pathologies, neurofibrillary tangles composed of hyper phosphorylated tau, and extracellular senile plaques composed of amyloid beta (Aβ). The immune response to these pathologies, neuroinflammation is now appreciated to be involved in disease progression. Regulated forms of neuroinflammation are viewed as protective, and indeed required for homeostatic function. In contrast, a dysregulated form of this process is present in AD^2^. This dysregulated form is now recognised as a contributor to AD pathogenesis.

Fundamental to this neuroinflammation are microglia, the resident innate immune cells within the central nervous system (CNS). Critically, microglia mount the initial neuroinflammatory response and further maintain it^3^. Upon recognition of noxious stimuli, microglia release a number of cytokines including interleukin (IL) 1β, IL6, and tumor necrosis factor alpha (TNFα) to create an inflammatory microenvironment^4^. This is seen in conjunction with chemokine secretion to recruit additional microglia. Microglia also have a number of roles outside immune-related functions. In particular, microglia are often viewed as macrophages of the CNS due to a shared hierarchal lineage and roles in phagocytosis^5^.

Due to diverse roles, microglia adopt a number of varied phenotypes with differential corresponding functions throughout the CNS. As such, large efforts have been made to better phenotypically characterize microglia under both normal and AD settings, primarily through transcriptomic-based approaches^6,7^. In the 5× familial AD mouse model, a single cell ribonucleic acid (RNA) -seq approach was used to investigate all immune cells, from which a unique population termed disease-associated microglia (DAM) was identified^8^. This population was associated with changes in phagocytosis and lipid metabolism, and primarily localized near Aβ plaques. DAM and DAM-like microglial populations have been further identified within other AD models^9^. In addition, investigations into microglial morphology have also been conducted. Structure follows function, and it is established that efficient and rapid morphological remodelling is required for microglia to perform varied tasks^10^. Recent work has also identified other brain residing macrophages that reside between the barriers of the brain^11^. Under certain conditions, these cells can enter the parenchyma and contribute to the overall pool of myeloid cells and may also contribute to phenotypical differences.

Dysregulation of microglial function is seen throughout AD. Genome wide association studies (GWAS) identify a number of coding variants highly expressed in microglia^12^. Of the GWAS identified risk factors, cluster of differentiation (CD) 33 and triggering receptor expressed on myeloid cells 2 (TREM2) have been shown to be involved in phagocytosis and response to stimuli when further examined both *in vitro* and *in vivo*^4,13,14^. Microglia harvested from aged dual amyloid precursor protein/presenilin 1 (APP_swe_PS1_ΔE9_) model of AD also show decreased expression of Aβ binding receptors and Aβ degrading enzymes^15^.

Similar to microglia, the type-I interferon (IFN) cytokine family are also critical in neuroinflammation. Named for their capacity to “interfere” with viral replication, IFNs are now recognised to have distinct roles outside of infection^16^. There are two predominant types of type-I IFNs, α with 14 subtypes, and the single subtype β^17^. The type-I IFNs signal through their cognate receptor, the interferon α/β receptor (IFNAR) which is composed of two subunits, IFNAR1 and IFNAR2. Ablation of the IFNAR1 subunit alone has been demonstrated to abrogate conventional signalling^18^. Downstream signalling cascades involve induction of a number of signal inducer and transduction complexes and interferon regulatory factors (IRFs). These proteins all mediate different effects. IRF9 is critical for downstream mediation, whilst IRF7 is critical for the induction of further type-I IFNs^19,20^. These lead to transcription of a wide array of interferon-stimulated genes (ISGs). However, the exact role of type-I IFNs within the CNS remains poorly understood^21–23^. Increased levels of IFNα and a number of ISGs are seen in individuals in both Aicardi-Goutières syndrome and systemic lupus erythematosus, demonstrating a link between type-I IFNs and CNS disorders^24–26^. Type-I IFNs are indeed able to regulate levels of IL1β, IL6 and TNFα which are consistently upregulated within AD^3,27–29^.

Of critical note however, are the observed links between microglia and type-I IFNs^30^. Blockade of type-I IFN signalling in a murine lupus model alters both the number of CD68 positive reactive microglia and microglial engulfment of synapses^26^. Furthermore, IRF7 has been shown to be required for early-stage microglial development^11^. Within AD, a number of transcriptomic studies have shown that type-I IFNs are critical modulators of specific microglial populations^8,31,32^. A meta-analysis of microglial transcripts from 69 individual disease states, including AD, revealed a co-regulated IFN gene set across all disease states observed^7^. Whilst these studies have been able to demonstrate an association, the functional implications of microglial type-I IFN signalling within the context of AD are yet to be fully explored.

We posit that it is microglial type-I IFN signalling that contributes to neuroinflammation in AD, and that type-I IFN signalling is involved in phenotypical and functional change in microglia. We make use of murine models lacking IFNAR1 and subsequent type-I IFN signalling alongside APP_swe_PS1_ΔE9_ model of AD to achieve this. Here we are able to demonstrate for the first time that type-I IFN is involved in phagocytosis and alters the microglial response to Aβ_1–42_. Lack of type-I IFN signalling also alters microglial morphology in APP_swe_PS1_ΔE9_ mice. Collectively, these results are the first to elucidate the precise phenotypic and functional role of type-I IFN signalling in microglia and provide a baseline for future bodies of work.

## Results

### Treatment of microglial cells with type-I IFN inhibits phagocytosis

**Figure 1:**
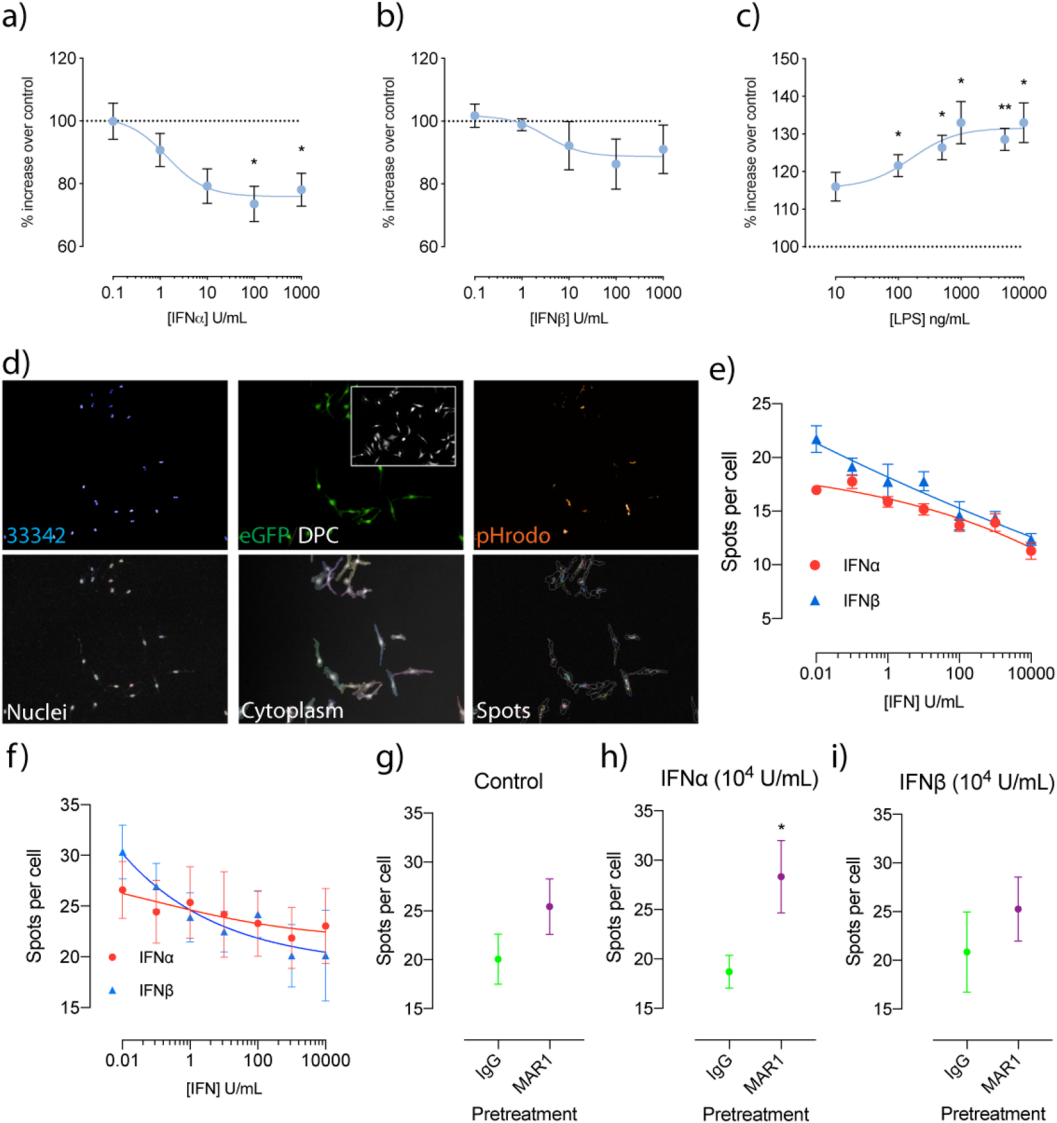
Treatment with IFN inhibits phagocytosis in both BV-2 and primary CX3CR1^eGFP/+^ primary microglia, with IFNα inhibition reversible with an anti-IFNAR1 neutralising antibody. Phagocytosis was determined using pHrodo *E. coli* fluorescent particles and is represented as % increases over untreated samples following varying concentrations of (**a**) IFNα, (**b**) IFNβ and (**c**) LPS (*p < 0.05, **p < 0.01, 1-way ANOVA, Dunnett’s multiple comparisons test, n = 5–6). (**d**) Workflow of imaging analysis for pHrodo *E.coli* treatments for both BV-2 and CX3CR1^eGFP/+^ primary microglia. Analysis of spots per cell following varying concentrations of IFN in (**e**) BV-2 cells and (**f**) primary CX3CR1^eGFP/+^ microglia. Analysis of spots per cell following IgG and MAR1 pre-treatment after which cells were exposed to (**g**) control (**h**) 10^4^ U IFNα and (**i**) 10^4^ U IFNβ (*p < 0.05, Students unpaired t-test, n = 5–6). Data is expressed as mean ± SEM.

Recently, type-I IFNs have been reported to be involved in the modulation of microglial phenotype. To investigate how type-I IFN stimulation directly affects microglial function, a phagocytosis assay utilizing pHrodo *E. coli* bioparticles was performed with immortalised BV-2 murine microglial-like cells. Dose response curves revealed that IFNα significantly decreased phagocytosis at concentrations of 100 (untreated: 100 ± 0% vs. 100U: 73.57 ± 5.656%, p = 0.0297, n = 5) and 1000 units (untreated: 100 ± 0% vs. 1000U: 78.07 ± 5.215%, p = 0.0423, n = 5) (Fig. 1a). Regression analysis also showed a negative relationship between phagocytosis and concentration. IFNβ treatments showed a similar trend for decreased phagocytosis with increasing concentration, however this was not statistically significant (Fig. 1b). To compare this to a classical pro-inflammatory response, this assay was repeated with lipopolysaccharide (LPS) (10–10,000 ng/mL) as a stimulus (Fig. 1c). Contrasting to the type-1 IFNs, significant increases in phagocytosis were seen with LPS concentrations greater than 100 ng/mL (untreated: 100 ± 0% vs. 100 ng/mL: 121.6 ± 2.89%, p = 0.0157, 500 ng/mL: 126.4 ± 3.249%, p = 0.0124, 1000 ng/mL: 133 ± 5.99%, p = 0.0305, 5000 ng/mL: 128.5 ± 2.978%, p = 0.0077, 10,000 ng/mL: 133 ± 5.2961%, p = 0.0261). This suggests that type-I IFNs are indeed eliciting a unique functional effect on microglia that differs from a classical pro-inflammatory response. For increased sensitivity and to confirm these findings, we also performed experiments using a high content imaging platform. The workflow is described in Fig. 1d. Regression analysis showed a negative relationship for both IFNα and IFNβ treatments in number of spots per cell, confirming our initial findings. We then repeated these experiments using primary CX3C chemokine receptor 1 (CX3CR1^eGFP/+^) microglia. Similarly, we are able to show a negative dose-response relationship. To then investigate if this inhibition can be reversed, cells were pre-treated with either IgG controls or an anti-IFNAR1 (MAR1) antibody before treatment with either 10^4^ units IFNα/β. MAR1 was able to increases the number of spots per cell in IFNα treated cells (9.632 ± 4.023, p = 0.0436).

### Monomeric Aβ_1–42_ triggers a type-I IFN response in wild type microglia that is ameliorated in IFNAR1^−/−^ microglia

**Figure 2:**
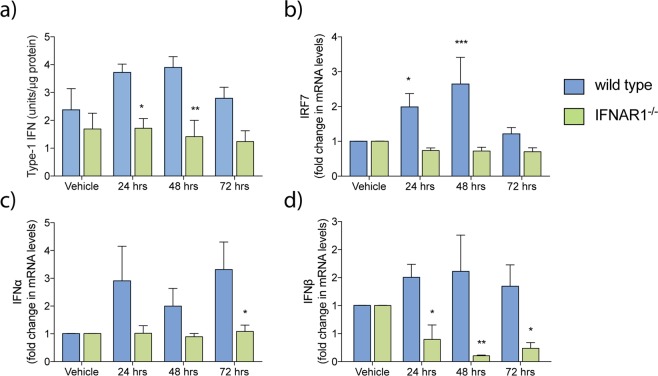
Aβ_1–42_ elicits a type-I IFN response in microglia that is ameliorated by removal of IFNAR1. Primary wild type and IFNAR1^−/−^ microglia were treated with 10 μM monomeric Aβ_1–42_ treatment (24–72 hours). (**a**) Collected media was analysed via reporter cell assay to measure levels of bioactive type-I IFN. Cells were then analysed via QPCR to measure transcript levels of (**b**) IRF7, as well as levels of both (**c**) IFNα and (**d**) IFNβ. Data is displayed as mean ± SEM (*p < 0.05, **p < 0.01, 2-way ANOVA, Sidak’s multiple comparisons test, wild type vs. IFNAR1^−/–^, n = 5–6).

We have previously demonstrated that ablation of type-I IFN signalling in APP_swe_PS1_ΔE9_ mice slowed cognitive decline and altered global glial phenotype^33^. Here, we focused specifically on examining the microglial response. To confirm that microglia do indeed mount a type-I IFN response, primary microglia of wild type and IFNAR1^−/−^ genotypes were subjected to monomeric Aβ_1–42_ treatment *in vitro*. Media was collected and analysed by a bioactive type-I IFN measurement assay (Fig. 2a). IFNAR1^−/−^ microglia showed decreased levels of bioactive type-I IFN compared to wild type at both 24 (wild type: 3.719 ± 0.299 U/mg total protein vs. IFNAR1^−/−^: 1.716 ± 0.585 U/mg total protein, p = 0.0427, n = 5–6) and 48 (wild type: 3.889 ± 0.391 U/mg total protein vs. IFNAR1^−/−^: 1.416 ± 0.585 U/ mg total protein, p = 0.0094, n = 5–6) hours after treatment.

We further investigated this type-I IFN response by analysing a number of type-I IFN related genes via quantitative polymerase chain reaction (QPCR), including IFNα, IFNβ, and IRF7 (Fig. 2b–d). Levels of IFNα were decreased in IFNAR1^−/−^ microglia at 72 hours only (wild type: 3.315 ± 0.992 fold vs. IFNAR1^−/−^: 1.087 ± 0.225 fold, p = 0.0322, n = 4–5). IFNβ was decreased at all time points measured (24 hours - wildtype: 1.503 ± 0.234 fold vs. IFNAR1^−/−^: 0.397 ± 0.258 fold, p = 0.0489, 48 hours – wild type: 1.609 ± 0.648 vs IFNAR1^−/−^: 0.105 ± 0.013, p = 0.0042, 72 hours – wild type: 1.345 ± 0.381 vs. IFNAR1^−/−^: 0.238 ± 0.104, p = 0.0486, n = 5–6). IRF7 was also decreased at 24 (wild type: 2.196 ± 0.217 vs. IFNAR1^−/−^: 0.723 ± 0.157, p = 0.0033, n = 5–6) and 48 hours (wild type: 2.769 ± 0.709 vs. IFNAR1^−/−^: 0.773 ± 0.141, p < 0.0001, n = 5–6). Levels of IRF3 remained unchanged between genotypes. This data suggests that the type-I IFN response induced by Aβ is IFNα, IFNβ and IRF7 mediated.

### IFNAR1^−/−^ microglia exhibit an altered inflammatory profile following monomeric Aβ_1–42_ treatment

**Figure 3:**
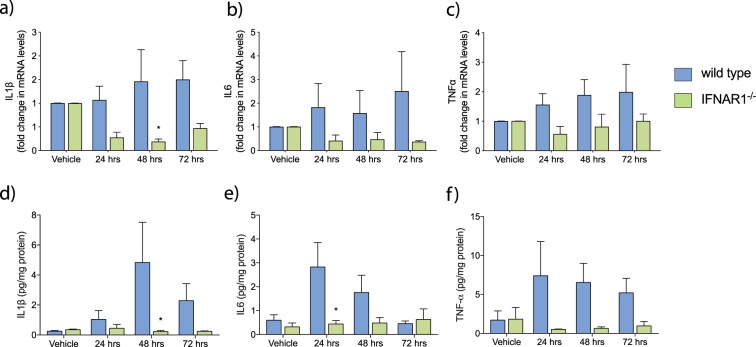
The inflammatory response to Aβ_1–42_ is attenuated in IFNAR1^−/−^ primary microglia. Transcript levels of (**a**) IL1β, (**b**) IL6 and (**c**) TNFα were analysed by QPCR analysis following 10 μM monomeric Aβ_1–42_ treatment (24–72 hours). Collected media was also analysed via ELISA for the corresponding cytokines (**d**) to (**f**). Data is displayed as mean ± SEM (*p < 0.05, 2-way ANOVA, Sidak’s multiple comparisons test, wild type vs. IFNAR1^−/–^, n = 5).

To further investigate the inflammatory response in IFNAR1^−/−^ microglia in response to Aβ treatment, levels of IL6, IL1β and TNFα were determined by QPCR. These cytokines have been shown to be critical in the neuroinflammatory response and are elevated in AD patients^3^. Transcript levels of IL1β (Fig. 3a) were significantly decreased in IFNAR1^−/−^ microglia at both 48 (wild type: 1.462 ± 0.669 fold vs. IFNAR1^−/−^: 0.174 ± 0.048 fold, p = 0.0076, n = 6) and 72 hours (wild type: 1.502 ± 0.399 fold vs. IFNAR1^−/−^: 0.383 ± 0.113 fold, p = 0.0245, n = 6) following Aβ treatment. No significant changes were detected in transcripts for either IL6 (Fig. 3b) or TNFα (Fig. 3c).

To confirm if these changes in messenger RNA (mRNA) expression translated to alterations in protein levels, enzyme-linked immunosorbent assays (ELISA) were performed on media from these same samples. Due to differences in purity between commercially available Aβ_1–42_, all results are expressed as a ratio compared to total media protein levels as measured by Bradford analysis. Between genotypes, significant differences were observed in levels of IL6 (Fig. 3d) at 24 hours (wild type: 2.83 ± 1.026 pg/mg total protein vs. IFNAR1^−/−^: 0.44 ± 0.144 pg/mg total protein, p = 0.0164, n = 5–6) and IL1β (Fig. 3e) at 48 hours (wild type: 4.83 ± 2.682 pg/mg total protein vs. IFNAR1^−/−^: 0.228 ± 0.077 pg/mg total protein). TNFα measurements showed a trend for decreased levels in IFNAR1^−/−^ microglia across all observed timepoints (Fig. 3f). This further confirms the pro-inflammatory response elicited by Aβ_1–42_ *in vitro* and identifies type-I IFNs as potential regulators of this response.

### APP_swe_PS1_ΔE9_ × IFNAR1^−/−^ microglia exhibit increased phagocytic capacity towards FITC conjugated Aβ_1–42_, but not FITC Aβ_42–1_

**Figure 4:**
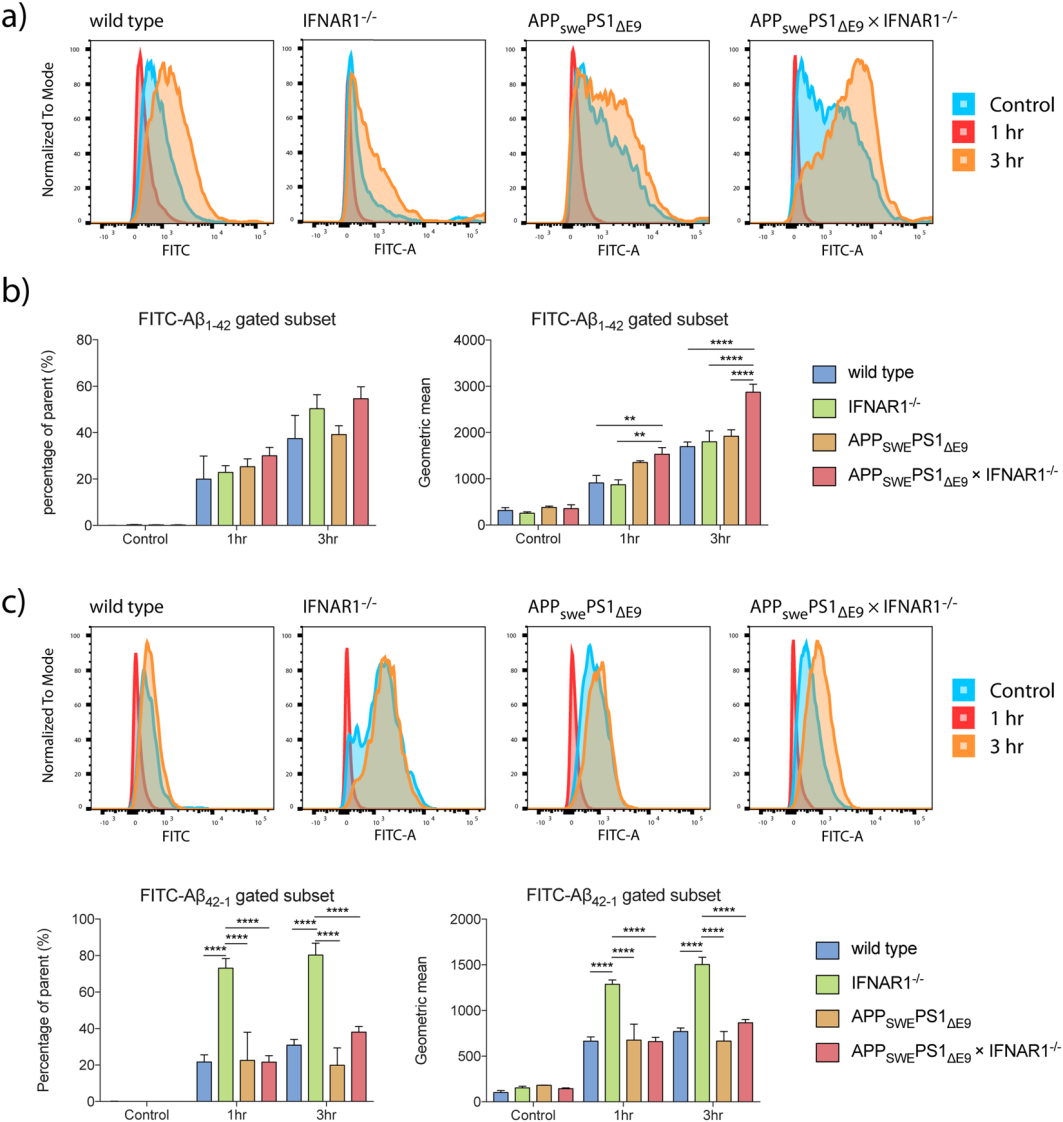
APP_swe_PS1_ΔE9_ × IFNAR1^−/−^ exhibit enhanced phagocytosis towards FITC-Aβ_1–42_, but not the reverse peptide FITC-Aβ_42–1_. Cells were treated with either FITC conjugated Aβ_1–42_ or the reverse peptide Aβ_42–1_ for 1 and 3 hours before being measured by flow cytometry. Representative distributions for each genotype for Aβ_1–42_ are shown in (**a**). Measures of percentage of parent and geometric mean are shown for 1–42 in (**b**). Representative distributions for each genotype for Aβ_42–1_ are shown in (**c**). Measures of percentage of parent and geometric mean are shown for 42–1 in (**d**). Data is displayed as mean ± SEM (****p < 0.0001, **p < 0.01, 2-way ANOVA, Sidak’s multiple comparisons test, wild type vs. IFNAR1^−/−^ vs. APP_swe_PS1_ΔE9_ vs. APP_swe_PS1_ΔE9_ × IFNAR1^−/−^, n = 3–5).

To further investigate the role between type-I IFN signalling and microglial function, wild type, IFNAR1^−/−^, APP_swe_PS1_ΔE9_ and APP_swe_PS1_ΔE9_ × IFNAR1^−/−^genotypes were treated with fluorescein isothiocyanate (FITC) conjugated Aβ_1–42_ peptide (2 mg/mL) for 1 and 3 hours, after which cells were analysed by flow cytometry. All genotypes showed similar percentage levels of FITC positive cells over 1 and 3 hours (Fig. 4e). However, the mean fluorescent intensity as assessed through geometric mean showed a marked increase in the APP_swe_PS1_ΔE9_ × IFNAR1^−/−^ microglia compared to all genotypes (1 hour: APP_swe_PS1_ΔE9_ × IFNAR1^−/−^: 1532.400 ± 143.406 vs. wild type: 911 ± 159.868, vs. IFNAR1^−/−^: 870.00 ± 107.699, p < 0.01, APP_swe_PS1_ΔE9_ × IFNAR1^−/−^ vs. all genotypes, n = 3–6, 3 hours: APP_swe_PS1_ΔE9_ × IFNAR1^−/−^: 2870.600 ± 175.241 vs wild type: 1695.250 ± 98.447, vs. IFNAR1^−/−^: 1801.66*7* ± 135.451, vs. APP_swe_PS1_ΔE9_: 1923.000 ± 135.451, p < 0.0001, APP_swe_PS1_ΔE9_ × IFNAR1^−/−^ vs. all genotypes, n = 3–6) (Fig. 4f). This data demonstrates that on an individual cellular basis, APP_swe_PS1_ΔE9_ × IFNAR1^−/−^ microglia have an enhanced ability to phagocytose FITC conjugated Aβ_1–42_. Interestingly, this is unique to these APP_swe_PS1_ΔE9_ × IFNAR1^−/−^ microglia, with IFNAR1^−/−^ microglia alone showing no differences.

To confirm if this finding was specific to Aβ or an increase in overall phagocytic ability, we then performed the same experiment using the reverse peptide FITC Aβ_42–1_. Interestingly, APP_swe_PS1_ΔE9_ × IFNAR1^−/−^ microglia showed no similar increases in uptake, but rather IFNAR1^−/−^ microglia showed significant increases in both percentage of parent (1 hour: IFNAR1^−/−^: 73.100 ± 5.254 vs. wild type: 21.700 ± 3.889, vs. APP_swe_PS1_ΔE9_: 22.627 ± 15.448, vs. APP_swe_PS1_ΔE9_ × IFNAR1^−/−^: 21.620 ± 3.565, p < 0.0001, IFNAR1^−/−^ vs. all genotypes, n = 3–5, 3 hours: IFNAR1^−/−^: 80.300 ± 6.585 vs. wild type: 30.933 ± 3.203, vs. APP_swe_PS1_ΔE9_: 19.957 ± 9.466, vs. APP_swe_PS1_ΔE9_ × IFNAR1^−/−^: 38.100 ± 3.102, p < 0.0001, IFNAR1^−/−^ vs. all genotypes, n = 3–5) and geometric mean (1 hour: IFNAR1^−/−^: 1287.100 ± 47.548 vs. wild type: 664.333 ± 46.423, vs. APP_swe_PS1_ΔE9_: 676.333 ± 173.680, vs. APP_swe_PS1_ΔE9_ × IFNAR1^−/−^: 659.000 ± 48.461, p < 0.0001, IFNAR1^−/−^ vs. all genotypes, n = 3–5, 3 hours: IFNAR1^−/−^: 1504.000 ± 79.618 vs. wild type: 770.333 ± 38.176, vs. APP_swe_PS1_ΔE9_: 665.333 ± 105.035, vs. APP_swe_PS1_ΔE9_ × IFNAR1^−/−^: 866.000 ± 35.940, p < 0.0001, IFNAR1^−/−^ vs. all genotypes, n = 3–5) at both 1 and 3 hours.

### Plaque burden in 9 and 13-month APP_swe_PS1_ΔE9_ × IFNAR1^−/−^ mice is unchanged

**Figure 5:**
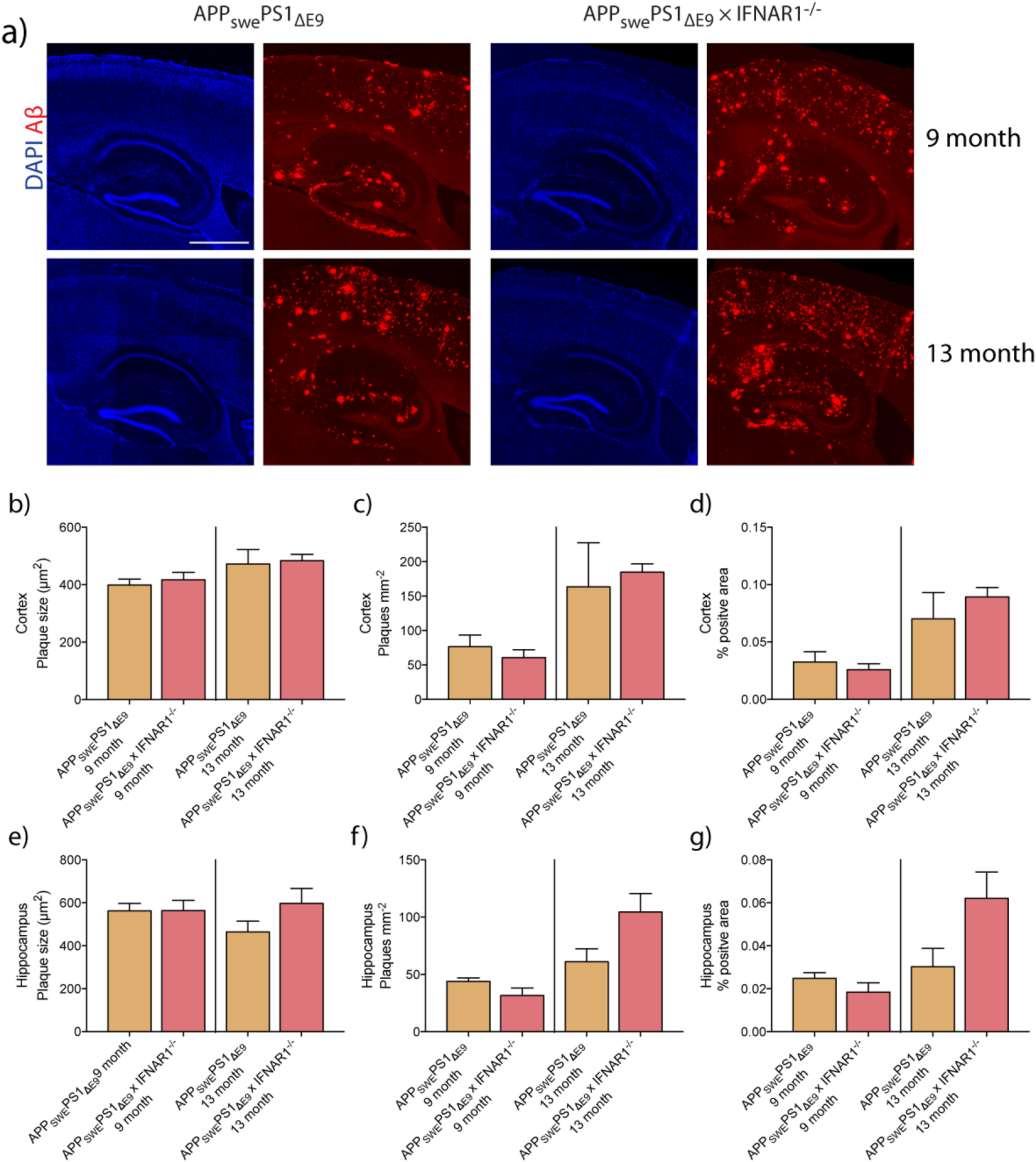
Aged 9 and 13-month APP_swe_PS1_ΔE9_ × IFNAR1^−/−^ brains exhibit unaltered Aβ burden compared to APP_swe_PS1_ΔE9_ mice. Brain sections from 9 and 13-month old APP_swe_PS1_ΔE9_ and APP_swe_PS1_ΔE9_ × IFNAR1^−/−^ were stained with anti-Aβ antibodies for automated plaque analysis. Representative hippocampal and cortical sections for each age of APP_swe_PS1_ΔE9_ and APP_swe_PS1_ΔE9_ × IFNAR1^−/−^ are shown in (**a**). Measurements of (**b**) cortical plaque size, (**c**) cortical plaque burden and (**d**) plaques per mm^2^ are shown. Measurements of hippocampal measures for each are shown in (**e**) to (**g**) respectively. Data is displayed as mean ± SEM, n = 3–6). Scale bar = 2 mm.

To investigate whether the observed increased Aβ phagocytosis translated *in vivo*, we performed immunofluorescence analysis of Aβ plaques in both 9 and 13-month APP_swe_PS1_ΔE9_ and APP_swe_PS1_ΔE9_ × IFNAR1^−/−^ mice. Sagittal brain sections were stained with an anti-Aβ antibody and imaged before being subjected to an automated analysis utilizing a watershed segmentation approach (Fig. 5b). Both APP_swe_PS1_ΔE9_ and APP_swe_PS1_ΔE9_ × IFNAR1^−/−^ mice show significant deposition throughout the brain. However, no significant difference was observed in either 9 or 13-month old mice when quantitation of plaque size, plaques per mm^2^ or % of plaque burden was performed in either the cortical or hippocampal areas (Fig. 5c–h). Both APP_swe_PS1_ΔE9_ and APP_swe_PS1_ΔE9_ × IFNAR1^−/−^ showed similar age-related increases in plaques. These findings suggest that removal of IFNAR1 does not alter plaque burden in either 9 and13-month old APP_swe_PS1_ΔE9_ mice.

### 13-month, but not 9 month, APP_swe_PS1_ΔE9_ × IFNAR1^−/−^ microglia adopt a stellate morphology

**Figure 6:**
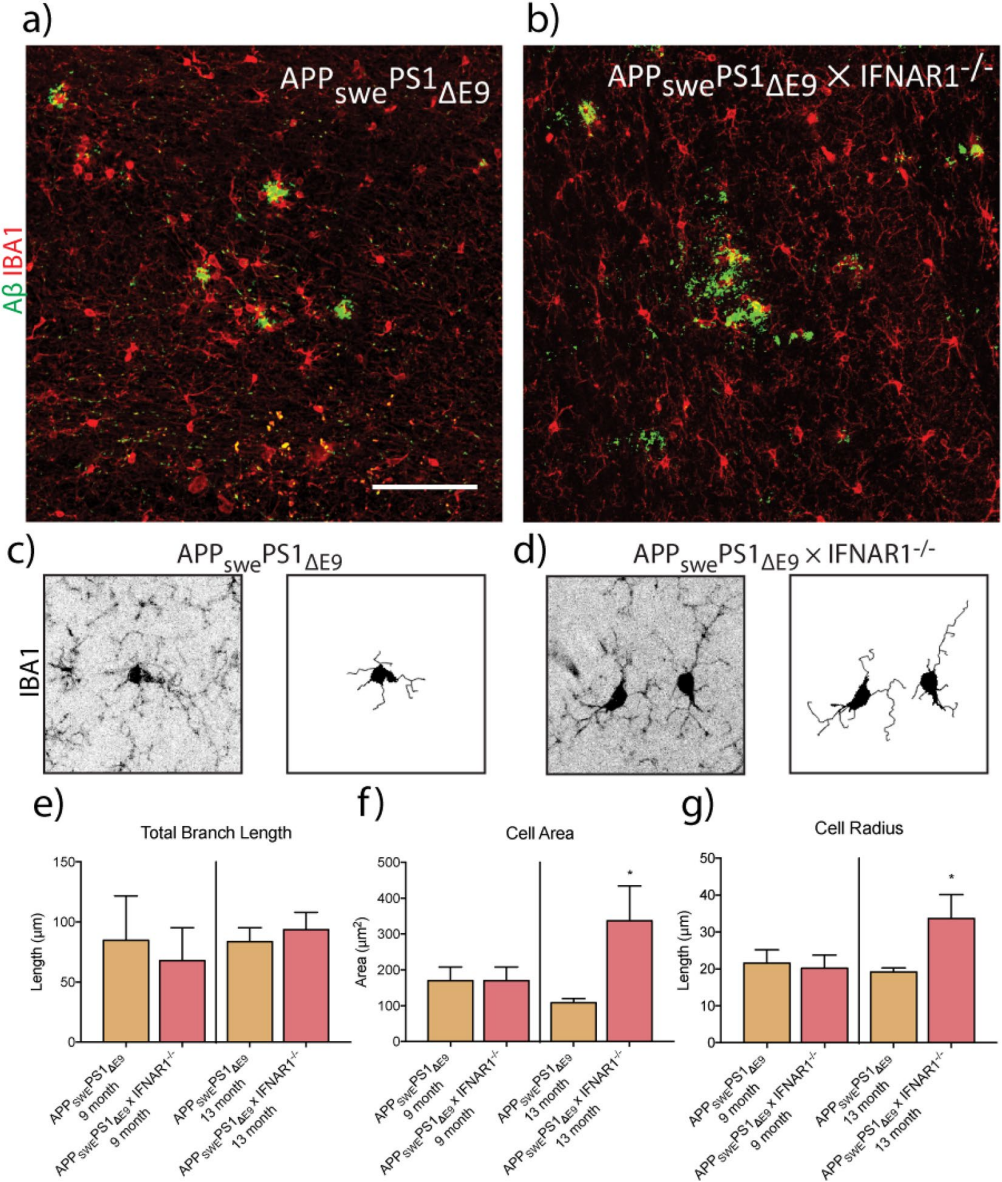
13-month APP_swe_PS1_ΔE9_ × IFNAR1^−/−^ microglia adopt a stellate-like morphology. 30 μm thick brain sections of 9 and 13-month old APP_swe_PS1_ΔE9_ and APP_swe_PS1_ΔE9_ × IFNAR1^−/−^ mice were stained with both IBA-1 and WO2 antibodies before 15 μm z-stacks were taken from of the hippocampal region. Representative images are shown for 13-month APP_swe_PS1_ΔE9_ and APP_swe_PS1_ΔE9_ × IFNAR1^−/−^ in (**a**) and (**b**) respectively. A minimum spanning tree algorithm was then employed to measure morphological characteristics with an overview of the skeletonising process in (**c**) and (**d**) for both genotypes respectively. Measurements are then shown for (**e**) total branch length, (**f**) cell area and (**g**) cell radius. Data is displayed as mean ± SEM (*p < 0.05, Students t-test, APP_swe_PS1_ΔE9_ vs. APP_swe_PS1_ΔE9_ × IFNAR1^−/−^, n = 3–8). Scale bar = 500 μm.

To further elucidate the effect that type-I IFN signalling and ageing has on microglial phenotype, we then performed a morphological analysis on both 9 and 13-month old APP_swe_PS1_ΔE9_ and APP_swe_PS1_ΔE9_ × IFNAR1^−/−^ mice (Fig. 6a,b). Here, we examined microglia not in direct contact with Aβ plaques, as the heightened clustering of microglia around plaques in aged mice poses difficulties in ascribing particular branches to individual microglia. Example traces are shown in Fig. 6c,d. At 13 months however, significant increases in the cell radius (APP_swe_PS1_ΔE9_: 19.14 ± 1.123 μm vs. APP_swe_PS1_ΔE9_ × IFNAR1^−/−^: 33.69 ± 6.472 μm, p = 0.0250, n = 6–8) (Fig. 6e) and cell area (APP_swe_PS1_ΔE9_: 108.6 ± 11.06 μm^2^ vs. APP_swe_PS1_ΔE9_ × IFNAR1^−/−^: 337.3 ± 96.5 μm^2^, p = 0.0179, n = 6–8) (Fig. 6f) in the APP_swe_PS1_ΔE9_ × IFNAR1^−/−^ microglia were detected. Combined with an unchanged level in branch length, these findings combined suggest that these microglia adopt a stellate like morphology. Such a morphology suggests an anti-inflammatory phenotype^4^.

## Discussion

Neuroinflammation is seen as a chronic and detrimental process within AD, and it is suggested that microglia are the critical cell type responsible for this response^34,35^. This is also seen in congruence with microglial dysfunction. As such the identification of key regulators of this neuroinflammatory process present as novel therapeutic targets^36^. Type-I IFNs are known to act as “master regulators” of the innate immune response and are able to regulate levels of IL1β, IL6 and TNFα which are consistently upregulated within AD brains^3,27–29^. Recent work investigating microglial phenotype has identified type-I IFNs as regulators of unique and conserved microglial populations in both ageing and disease^8,31,32,37^. Here, we further examine the modulation of these phenotypic microglia by the type-I IFNs, critically focussing on investigating their functional roles.

We firstly identified that type-I IFN treatment decreases the ability of microglial phagocytosis in a dose-dependent manner. Through genetic abrogation of type-I IFN signalling by targeting its receptor IFNAR1, we identify that IFNAR1^−/−^ primary microglia exhibit a decreased IL1β, IL6 and TNFα response to monomeric Aβ_1–42_, and that targeting IFNAR1 within primary microglia isolated from the APP_swe_PS1_ΔE9_ AD model leads to an enhanced ability to phagocytose FITC Aβ_1–42_. These aged APP_swe_PS1_ΔE9_ × IFNAR1^−/−^ mice also show altered microglial morphology *in vivo*.

We have previously reported that ablation of type-I IFN signalling within the APP_swe_PS1_ΔE_ AD model is neuroprotective^33^. In this model, reduced type-I IFN signalling was associated with an attenuated whole-brain inflammatory profile and a rescue in cognition as assessed via the Morris water maze. The cell-specific contributions that resulted in this altered response were unknown. This suggested that microglia were indeed the cell type responsible.

Microglial function follows phenotype, and phagocytosis is recognised as a critical function of microglia^38^. We first employed a phagocytosis assay on microglial cells to investigate how type-I IFN stimulation affects this process. A negative phagocytic dose-response relationship was observed in cells treated with IFNα, with a trend for a decrease in IFNβ also seen. Previous work investigating IFN and phagocytosis has shown differing results. In an autoimmune encephalomyelitis model of multiple sclerosis, IFNβ was shown to increase microglial phagocytosis of myelin^39^. Furthermore, increased myelin was observed within the brains of IFNAR1^−/−^ knockout mice. Phagocytosis is not limited to a single pathway, and both myelin and Aβ may indeed invoke separate cellular mechanisms that result in engulfment^40^. There may also be other kinetic measures that explain these differences which are masked in end-point assays. Investigation in macrophages and other mononuclear phagocytes also demonstrate a role for type-I IFNs in promoting phagocytosis^41–43^. Similarly, these studies also use different particles to measure phagocytosis. Regardless, further work is required to investigate these findings. Our results confirm work that type-I IFNs are involved in microglial function, with data demonstrating that IFNα in particular affects phagocytosis. Interestingly, these decreases contrasted to the increases seen with the pro-inflammatory LPS. To further investigate this finding, we also performed similar experiments using a high content imaging platform for increased sensitivity. We are able to confirm our initial findings with BV-2 cells. Levels of type-I IFNs are known to increase with normal ageing, with our study supporting previous data identifying an impaired phagocytic function of aged microglia^31,44^. We are able to demonstrate that type-I IFNs elicit differential functional effects on microglia when compared to classical inflammatory stimuli, suggesting that type-I IFNs are able to shift microglia to a unique phenotype. This is in line with recent findings identifying unique microglia populations within the CNS that are enriched with a number of IFN regulated genes^45^.

Consistent with our previous findings examining Aβ treatments on primary mixed glial cultures, our study confirmed that Aβ_1–42_ elicits a type-I IFN response in wild type microglia, with this response attenuated in IFNAR1^−/−^ microglia. Increases were seen in levels of bioactive type-I IFN at 24 and 48 hours between genotypes, which is mirrored in the mRNA transcripts of both IFNα and IFNβ. Within IFNAR1^−/−^ microglia transcript levels of IFNα were observed to remain constant, but IFNβ levels actively downregulated. This suggests differing roles for IFNα and IFNβ in AD, and that IFNα is the critical subtype that affects microglia within AD. Type-I IFNs are known to amplify their responses in an autocrine manner and elicit differing responses depending on both type and subtype^46^. In congruence, levels of IL1β and IL6 are actively decreased within these same IFNAR1^−/−^ microglia. This suggests that expression of these hallmark proinflammatory cytokines may be downstream of type-I IFN signalling. Furthermore, alteration of microglial phenotype through reduction of these hallmark pro-inflammatory cytokines is well established in shifting microglial function^47^. Levels of IRF7 were upregulated between wild type and IFNAR1^−/−^ genotypes at both 24 and 48 hours respectively. In primary macrophages, it has been reported that IRF7 is able to elicit differential type-I IFN responses, with IRF7 associated with an overall heightened response level^48^. Furthermore, IRF7 is known to be a major IRF induced downstream of type-I IFN responses and is critical in further induction of the IFN response^19^.

Phagocytosis is a critical functional process of microglia impaired in AD^3^. Here we observed that APP_swe_PS1_ΔE9_ × IFNAR1^−/−^ microglia, but not IFNAR1^−/−^ microglia alone, exhibited greater phagocytic ability towards FITC Aβ_1–42_. This was compounded by the increases in uptake on a per-cell basis of IFNAR1^−/−^ microglia of the reverse peptide, FITC Aβ_42–1_. Whether this result is a unique interaction between APP_swe_PS1_ΔE9_ and IFNAR1 or indeed due to other processes remains unknown, and as such warrants further investigation. One such explanation may be microglial “priming”, a concept explored within neuroinflammation and AD^49^. Levels of Aβ have been observed in culture from transfected cells that contain human APP, which may be responsible for alterations in microglial phenotype^50^. Bias for particular Aβ species may also be involved. The nature of FITC conjugation denotes that aggregation dynamics differ between it and unlabelled peptides^51^. This oligomerisation bias may also explain the observations *in vivo*, with APP_swe_PS1_ΔE9_ × IFNAR1^−/−^ mice showing decreases in monomeric levels of Aβ_1–42_ only^33^. The observed increases in the IFNAR1^−/−^ microglia may indeed be due to differential phagocytic processes. Critically, the reverse peptide has differential aggregation dynamics when compared to the forward peptide sequence^52^. Multiple receptors are involved in microglial phagocytosis that differ in alterations in cytoskeletal elements, phagosome maturation and inflammatory responses in response to binding^38,40^. It is important to note that previous *in vitro* work investigating immune-related gene knockouts and fluorescent Aβ phagocytosis have not considered the role of their respective AD genotype^13,53^.

Due to these observed differences in uptake of Aβ_1–42_, we then examined Aβ plaque burden as a surrogate measure of *in vivo* microglial phagocytosis. Critically, we expand our analysis from our previous work. We observed no differences between genotypes, which follow our previous findings^33^. It is well established that plaque burden is not a measure of disease state, as levels do not correlate with cognition. Furthermore, plaque reduction does not alter pathology and cognitively normal individuals containing plaques within their brains^54–56^.

We further investigate these mice though use of a sophisticated, and critically, verified, algorithm to examine a number of morphological features^57^. Increased cellular radius and diameter without increases in branch length indicate that APP_swe_PS1_ΔE9_ × IFNAR1^−/−^ microglia adopt a stellate-like morphology. The exact nature of how this relates to underlying phenotype however remains unknown. Classically, ramified-like morphologies have classically been used to identify “quiescent” or “resting” microglial states and amoeboid-like morphologies for “reactive” states^58^. Similar morphologies can however exhibit altered phenotypes^59^. As such, the extent to which this classic paradigm holds true remains unknown. However, quantified morphological analyses add an additional trait to further our understanding of microglia. Further work is required to link various morphological features and microglial functions, both in normal and disease states, as well as across ageing.

Our observations combined with our *in vitro* data suggest that these microglia possess an altered anti-inflammatory phenotype. The observation of altered morphology at 13 but not 9 months of age in APP_swe_PS1_ΔE9_ × IFNAR1^−/−^ microglia is notable and calls for further examination. It is established that APP_swe_PS1_ΔE9_ mice begin to show a decreased cognitive phenotype at 6 months, and as such 13-months is an advanced stage for disease^60^. This heightened disease state may overcome protective effects due to loss of IFNAR1^−/−^ as we have previously observed, in turn altering microglial phenotype and subsequent morphology. Future work will focus on the direct analysis of microglia isolated from these mice with a transcriptomic based approach utilised, similar to that reported in other related studies focused on microglial phenotypes^8,31^. This combined with morphological data will allow for deeper insight into how the type-I IFN signalling pathway is involved in regulating microglial function.

## Conclusions

These results demonstrate that type-I IFNs are involved in the modulation of both microglial phenotype and function. This work further expands on emerging data demonstrating a link between microglia, type-I IFNs and AD. Type-I IFNs present as a much-needed novel therapeutic target for the management of AD.

## Methods

### Animals

C57BL/6J wild type mice were sourced from the Animal Resource Centre. IFNAR1^−/−^ mice were initially generated by Hwang, *et al*.^18^. APP_swe_PS1_ΔE9_ transgenic mice were originally sourced from JAX^61^. APP_swe_PS1_ΔE9_ transgenic mice lacking IFNAR1^−/−^ were generated by Minter *et al*.^33^. All mice were housed in sterile micro-isolator cages and fed ad-libitum. All animal procedures were performed in accordance with the University of Melbourne animal care committee’s regulations and conducted in compliance with the Australian National Health and Medical Research Guidelines. All experiments were approved by the University of Melbourne Animal Ethics Committee (Ethics ID: 1613905).

### Genotyping

Genotyping was performed commercially using Transnetyx™.

### pHrodo phagocytosis assay

BV-2 microglial cells (a generous donation from Dr. Sherif Boulos, Western Australian Neuroscience Research Institute) were plated within black flat-bottomed 96 well plates (Corning, 3603) at 2.5 × 10^3^ cells per well. Fresh culture media was applied to cells (Dulbeccos modified eagle medium (DMEM)) (10569–044, Gibco), 5% FBS, 1% penicillin/streptomycin) 24 hours before cells were incubated with serum free DMEM containing various concentrations of IFNα, IFNβ and LPS. Cells were then incubated for 1 hour at 37 °C and 5% CO_2_ before each well was rinsed with 100 μl of DMEM before media was replaced with DMEM containing pHrodo (ThermoFisher, P35361) diluted 1:30. Plates were viewed under a fluorescence microscope to ensure phagocytosis had occurred before being read using a Flexstation II (Molecular Devices) or Operetta High Content Imaging machine (Perkin Elmer).

### Mixed cortical and hippocampal glial isolation

Mixed cortical and hippocampus glial cultures were isolated from P0-P1 pups as described previously^33^. Briefly, cortices and hippocampal structures were isolated, and meninges then surgically removed. This cleaned tissue was then placed into a solution of hanks buffered saline solution (HBSS) containing trypsin ethylenediaminetetraacetic acid (EDTA) (1 × final concentration, 59418 C, Sigma) and deoxyribonuclease (DNAse) (1 mg/mL, D5025, Sigma) for 15 minutes, after which the supernatant was collected. Remaining undigested tissue was then subject to a second round of digestion before the supernatants were combined. Cells were plated at a density of 1.25 × 10^4^ cells/mL in culture medium (DMEM containing 20% foetal bovine serum (FBS), 1% penicillin/streptomycin). Media was replaced at days 2, 7 and 14 after which the glial cells formed a monolayer.

### Microglial isolation

Microglia were isolated from mixed glial cultures at 18 days *in vitro*. Isolation was performed using the Saura, *et al*.^62^ mild trypsinization method and fluorescence activated cell sorting (FACS) for CX3CR1^eGFP/+^ microglia. Conditioned media was collected from each individual plate before undergoing a wash with DMEM. Media was then replaced with a 1:1 mixture of DMEM and 0.25% trypsin EDTA (25200-072, Gibco) and left for 30 minutes. Once detachment of the upper glial layer was confirmed, cells were again washed with DMEM before the collected conditioned media was returned to the individual plates. Cells were given a further 2 days incubation before use in experiments. Microglial purity was approximately 95% as analysed periodically through flow cytometry using a FITC conjugated CD11b antibody (130-113-796, Miltenyi Biotech).

### Amyloid-β preparation and treatment

The Aβ_1–42_ peptide (A-42-T-1, GenicBio) was reconstituted in 1,1,1,3,3,3-hexafluoro-2-propanol (HFIP) (1 mg/mL, 105228, Sigma) before combined monomerization and lyophilisation in a Heto Maxi Dry Lyo DC40 Dynavac freeze-drying centrifuge (12000 × G, room temperature, 30 minutes). Peptide was then dissolved in ice-cold 5 mM NaOH (in PBS), after which final working concentrations were measured via absorbance spectroscopy at 214 nm on a NanoDrop 3000 spectrophotometer.

### FITC-conjugated amyloid-β treatment

Cells were treated with serum free DMEM containing 2 mg/mL of FITC conjugated Aβ_1–42_ or Aβ_42–1_ (1% sodium azide, PBS) for 1 and 3 hours respectively. Cells were rinsed before being scraped in PBS and centrifuged (1500 × G, 5 minutes, room temperature) to form a pellet. Resulting pellets were then resuspended in FACS buffer (1% BSA, 100 ng/mL 4′,6-diamidino-2-phenylindole (DAPI), in PBS) and transferred into 5 mL cytometer tubes. Samples were read on a BD Fortessa flow cytometer.

### B16 IFN assay

B16-Blue cells (bb-ifnt1, InvivoGen) were used to measure bioactive type-I IFN in collected media as per manufacturers instructions. Briefly, cells were cultured in T75cm^2^ flasks in Roswell Park Memorial Institute 1640 (21870–092, Gibco) medium containing 5% FBS, 1% penicillin/streptomycin and zeocin (100 μg/ml). Cells were then plated in a 96 well plate at 75,000 cells/well. Collected media was added to these cells for 24 hours alongside IFNα (0–1000 U/mL) to generate a standard curve. QUANTI-Blue was then added in 1:1 v/v ratio for a further 24 hours. Following this, 200 μL of media was transferred into a 96 well plate and absorbance was measured on a Multiskan Ascent plate reader.

### ELISA

Murine Il1β (559603, BD Biosciences) IL6 (555240, BD Biosciences), and TNFα (DY410, R&D Systems) were used to detect protein levels in collected media as per manufacturer’s instructions. Briefly, 96-well plates were coated overnight in capture antibodies diluted in assay diluent (1:1000) at 4°. Plates were washed thrice, then blocked in assay diluent. Wells were then filled with either recombinant standards or 100 μL media from collected samples (2 hours, room temperature. Plates were then washed 5 times after which 100 μL working detector (detection antibody + SAv-HRP reagent) was added (1 hour, room temperature). Plates were then washed 7 times and a 1:1 mixture of hydrogen peroxide and 3,3′, 5,5′ tetramethylbenzidine was added (30 minutes, room temperature). The reaction was then stopped using sulphuric acid (160 mM) and absorbance measured at 450 nm in a Multiskan Ascent spectrophotometer (Thermo Scientific)

### RNA isolation

All samples underwent a modified RNA isolation protocol using both Trizol® (Invitrogen) and the illustra RNAspin mini kit (25050071, GE Health Sciences). Cultured cells were rinsed once in DMEM before being scraped in 1 mL Trizol® and transferred into a fresh autoclaved RNAse/DNAse free tube. Samples were then homogenized using a 23 g needle and 1 ml syringe. 0.2 mL chloroform was then added and samples shaken vigorously for 15 seconds before incubation for 3 minutes at room temperature. Samples were centrifuged (12000 × G, 15 minutes, 4 °C), after which the clear supernatant was collected and transferred into a new ribonuclease (RNAse)/DNAse free tube. 70% ethanol was then added to the supernatant at a ratio of 1:1, after which the mixture was placed onto the illustra RNAspin collection column. Isolation was then performed as per manufacturer’s instructions. Sample concentration and purity was assessed using a NanoDrop 1000 spectrophotometer. (ThermoScientific). All samples measured underwent on-column DNAse treatment and had 260/280 ratios above 2.

### Reverse transcription

1–2 μg of total RNA was reverse transcribed using the High Capacity complementary DNA (cDNA) Reverse Transcription Kit (4368814, Applied Biosciences) as per manufacturer’s instructions. Individual reactions were prepared, and PCR was performed in a thermal cycler (Eppendorf) under the following conditions: 10 minutes at 25 °C, 120 minutes at 37 °C, 5 minutes at 85 °C and 10 minutes at 4 °C. Each sample group also contained an additional reaction without the addition of reverse transcriptase enzyme (-RT). Final cDNA samples were diluted 1:10 for each 1 μg of initial RNA.

### QPCR

QPCR was performed within 384 well plates (4309849, Applied Biosystems) using a QuantStudio 6 PCR system (Applied Biosystems) with reactions performed in triplicate. Two detections system were used within this study: Taqman-based detection utilizing 5(6)-carboxyfluorescein (FAM) fluorescence reporting and oligomer-based detection utilizing fluorescent SYBR green DNA binding. For Taqman based detection all primers were purchased commercially (431182, Applied Biosystems), with details of each outlined in Table 1. Reactions were performed in 5 μL volumes and were composed of the following: 2 μL diluted cDNA, 0.25 μL Taqman primer, 0.25 μL RNAse DNAse free H_2_O and 2.5 μLm universal master mix reagent (SsoFast Probes, Biorad). For SYBR green detection, all oligomers were synthesized commercially (Geneworks, Integrated DNA Technologies) and our outlined in Table 2. Reactions were performed in 5μL volumes and were composed of the following: 2 μL diluted cDNA, 0.25 μL gene-specific forward primer, 0.25 μL gene-specific reverse primer and 2.5 μL fast SYBR green master mix (4385612, Applied Biosystems). The cycle threshold (Ct) values were normalized to a control gene (B2M) and analysed using the ΔΔCt method to generate fold change values.

**Table 1:** List of Taqman primers used in this study.

Gene	Species	Refseq	Amplicon length (bp)	Catalogue no.
B2M	Mouse	NM_009735.3	77	Mm00437762_m1
IRF7	Mouse	NM_001252600.1	67	Mm00516788_m1
		NM_001252601.1		
		NM_016850.3		
IFNβ	Mouse	NM_010510.1	69	Mm00439552_s1
Il1β	Mouse	NM_008361.3	63	Mm01336189_m1
IL6	Mouse	NM_031168.1	78	Mm00446190_m1
TNFα	Mouse	NM_001278601.1	81	Mm00443258_m1
		NM_013693.3

**Table 2:** List of SYBR primers used in this study.

Gene	Forward primer	Reverse primer(s)
B2M	GGCTCACACTGAATTCACCCCCAC	ACATGTCTCGATCCCAGTAGACGGT
IFNα	SAWCYCTCCTAGACTCMTTCTGCA	TATDTCCTCACAGCCAGCAG
		TATTTCTTCATAGCCAGCTG

### Immunofluorescence

Mice were anesthetized via intra-peritoneal injection of a combinatorial mixture of ketamine (100 mg/kg) and xylazine (10 mg/kg). Deep anaesthesia was confirmed through absence of paw withdrawal reflex. Mice then underwent cardiac perfusion with PBS to remove blood from the cerebral vasculature, after which 4% paraformaldehyde (PFA) was then delivered to fix the brain. Brains were then excised and placed into a solution of 4% PFA for 24 hours at 4 °C after which they were transferred into 30% sucrose solution for 24 hours at 4 °C for cryoprotection. Brains were then submerged in optimal cutting temperature media (OCT) (Sakura) within 2 cm^2^ plastic containers and frozen by immersion within an isopropanol dry-ice bath. 30 μm sections were cut sagittally on a cryostat from the initiation of the hippocampal structure to its completion. Sections were then placed into an individual well of a 24 well plate containing PBS for downstream staining. Collected sections were rinsed once in PBS for 5 minutes, after which they were simultaneously permeabilized and blocked by being placed in 200 μL of a solution containing 1.5% Triton-x (Sigma) in 10% goat block (G9023) in PBS (2 hours, room temperature, constant rocking at 20RPM). Sections were then washed thrice in PBS (room temperature, 4 minutes per wash) before incubation with primary antibodies (4 °C, overnight, constant rocking at 20RPM). Details of antibodies and concentrations used are listed in Table 3. Sections were then washed thrice (room temperature, 5 minutes per wash) before incubation with goat fluorescent secondary antibodies occluded from light (1:1000 dilution in PBS, room temperature, A-11012, A-11001, Invitrogen). Sections were washed thrice a final time (room temperature, 5 minutes per wash) before being rinsed in MilliQ H_2_O. Using a wide orifice pipette, sections were transferred onto slides and allowed to dry completely before coverslips were mounted using DAPI containing mounting media (H-1500, Vectashield). Images were obtained using the Zeiss Axio Observer.Z1 or Zeiss LSM 780 (Carl Zeiss imaging) inverted fluorescence microscopes with manipulation used only to increase contrast. Details of antibodies used are listed below in Table 3:

**Table 3:** List of antibodies used in this study.

Antibody	Origin	Concentration	Vendor	Catalogue no.
Anti-IBA1	Rabbit	1 in 200	Wako	019–19741
Anti-Aβ	Rabbit	1 in 500	Cell Signalling	#8243
WO2	Mouse	1 in 500	^63^	N/A
Alex Fluor 488 Goat anti-mouse	Goat	1 in 1000	Invitrogen	A-11001
Alex Fluor 594 Goat anti-rabbit	Goat	1 in 1000	Invitrogen	A-11012

### Plaque analysis

Entire brain sections were imaged on the Zeiss Axio Observer.Z1 wide-field microscope, after which associated Zeiss ZEN software was used to trace brain regions of interest. Final analysis was then done on ImageJ using the built-in watershed function.

### Morphological analysis

30 μm sagittal brain sections were collected approximately 1400 μm from midline. A single field was imaged in the CA1 region of the hippocampus with 15 μm z-stacks at 0.96 μm per slice, after which images then underwent maximum intensity projections before analysis. Images were analysed using a proprietary spanning tree path finding based algorithm with a minimum of 5 microglia per image analysed^57^. Trace images were then analysed on Matlab v2013b using built-in functions.

### Statistical analysis

All statistical analysis was performed using GraphPad Prism (Version 7). For pHrodo assay data, a 1-way analysis of variance (ANOVA) was performed followed by a Dunnett’s multiple comparisons post-hoc test. For bioactive IFN, QPCR, ELISA and FITC-Aβ data, a 2-way ANOVA was performed with a Sidaks multiple comparisons post-hoc test. Amyloid quantification data and morphological analysis utilized a Students t-test. All tests used considered a p < 0.05 statistically significant.

## Data Availability

Data from this study is included in this published article. Further information is available from the corresponding author on request.
